# Predicted visceral adiposity index in relation to risk of coronary heart disease and all-cause mortality: insights from NHANES

**DOI:** 10.3389/fendo.2023.1296398

**Published:** 2024-01-08

**Authors:** Yixing Luo, Xiangpeng Zhan, Yang Liu, Luyao Chen, Liang Zhu, Wenyao Cai

**Affiliations:** ^1^ Department of Gastroenterology, The First Affiliated Hospital, Jiangxi Medical College, Nanchang University, Nanchang, Jiangxi, China; ^2^ Department of Urology, The First Affiliated Hospital, Jiangxi Medical College, Nanchang University, Nanchang, Jiangxi, China; ^3^ Department of Cardiology, The Second Affiliated Hospital, Jiangxi Medical College, Nanchang University, Nanchang, Jiangxi, China; ^4^ Department of Cardiology, The First Affiliated Hospital, Jiangxi Medical College, Nanchang University, Nanchang, Jiangxi, China

**Keywords:** visceral adiposity index, coronary heart disease, NHANES, relationship, prognosis, all-cause mortality

## Abstract

**Background and aims:**

The Visceral Adiposity Index (VAI) is a straightforward and gender-specific marker that combines anthropometric measurements with lipid profiles. The objective of this study was to evaluate the relationship between VAI and coronary heart disease (CHD).

**Methods and results:**

The study examined data collected from adults during the NHANES 1999-2018 cycle. The analyses were weighted, and multivariable logistic regression models were employed to investigate the association between VAI and CHD. Additionally, subgroup analyses stratified by age were conducted. To evaluate the impact of VAI levels on survival outcomes, the study utilized the Kaplan-Meier method and performed the log-rank test to evaluate the survival outcome of participants with different VAI levels. The study findings revealed a significant association between VAI and CHD, indicating a non-linear relationship where an increase in VAI was associated with an elevated risk of CHD. High levels of VAI were linked to an increased prevalence of CHD (Q4 vs Q1, OR 1.50, 95% CI 1.12-2.01, P=0.01). Additionally, higher levels of VAI were associated with a poorer overall prognosis in terms of survival outcomes. There were no statistically significant differences in survival outcomes among the population with CHD.

**Conclusion:**

The results of this study highlighted a significant association between VAI and CHD, with a non-linear relationship observed. High VAI levels were associated with an increased risk of CHD and poor survival outcomes, emphasizing the importance of understanding and managing this risk factor, particularly in older age groups.

## Introduction

Coronary heart disease (CHD) was a complicated and chronic illness associated with high morbidity and mortality rates. It occurred primarily due to the development of atherosclerotic lesions in the coronary arteries, leading to stroke and myocardial infarction (MI) (1). The initiation of coronary atherosclerosis involved endothelial dysfunction and lipid buildup in the inner layer of the arteries. This progressed to the formation of fibrous and atherosclerotic plaques, eventually resulting in the development of composite plaques, including calcification. The stability of these plaques was closely linked to the occurrence of MI. Vulnerable plaques, known for their instability and prothrombotic nature, were prone to rupture. Inflammation played a critical role in the formation and rupture of these vulnerable plaques, as it could trigger blood clot formation, ultimately leading to MI (2). Several risk factors contributed to the development of CHD, such as dyslipidemia, hypertension, insulin resistance, hypercoagulability, and obesity (3). Among these factors, obesity gained significant attention for its involvement in CHD development. Throughout the 20th century, CHD became the leading cause of death in the US, resulting from reduced blood flow to the heart muscle caused by narrowed or blocked coronary arteries (4). Nevertheless, since the mid-1960s, mortality rates related to CHD had been decreasing. This decline could be attributed to advancements in medical and surgical treatments, as well as preventive measures like adopting healthier diets, managing hypertension, and controlling high cholesterol levels. The reduction in CHD mortality was a result of improved strategies for prevention, treatment, and management.

The prevalence of overweight and obesity had reached epidemic proportions in Western countries, becoming the second leading cause of preventable death after tobacco use (5, 6). Obesity was a significant independent risk factor for cardiovascular disease (CVD), including conditions like hypertension, coronary heart disease, atrial fibrillation, and heart failure. Although obesity contributed to several established CVD risk factors, it was observed that some types of CVD had a better prognosis in overweight or obese individuals compared to those with lower body weight. This counterintuitive phenomenon was often referred to as the “obesity paradox.” (7) The primary impact of obesity on CHD risk stemmed from atherogenic dyslipidemia and metabolic syndrome/diabetes mellitus. This was supported by evidence from the INTERHEART study (8), which involved the assessment of 30,000 patients in 52 countries. The research indicated that nearly 90% of the risk associated with acute myocardial infarction could be traced back to nine factors that can be modified. Among these factors, dyslipidemia emerged as the primary contributor, accounting for approximately half of the risk for developing acute MI. Visceral adipose tissue (VAT) served as a reliable marker for obesity in patients. Previous investigations consistently demonstrated elevated VAT in individuals with CHD (9). These studies typically employed computed tomography (CT) or magnetic resonance imaging (MRI) techniques to assess VAT levels. Additionally, researchers utilized the visceral adiposity index (VAI), which was calculated based on body mass index (BMI), waist circumference (WC), triglyceride levels (TG), and high-density lipoprotein cholesterol (HDL-c), as an alternative measure. Compared to CT or MRI methods, VAI offered a simpler, more cost-effective, and convenient approach to estimating VAT (10). Previous investigations also established a correlation between VAT and various conditions such as diabetes, hyperuricemia, metabolic syndrome, hypertension, atherosclerosis, and vascular calcification (3, 10, 11). The study by Marco C. Amato et al. suggested that VAI was a valuable indicator of “visceral adipose function” and insulin sensitivity, with its increase being closely associated with cardiovascular metabolic risk (12). Meanwhile, Yangchang Zhang et al. have found a correlation between VAI and cardiovascular and cerebrovascular diseases (13).

Therefore, the objective of this study was to evaluate the relationship between VAI and CHD among participants in the National Health and Nutrition Examination Survey (NHANES) from 1999 to 2018. Additionally, to assess the association of VAI with survival outcomes in both the general population and individuals with CHD.

## Method

### Study population

The NHANES study was a vital nationwide research project that utilized a comprehensive approach to recruit participants, guaranteeing a representative sample of the noninstitutionalized civilian population in the United States. It employed a multistage probability and oversampling design to ensure demographic diversity. Updated information from the study was regularly released every two years, and each participant’s data represented approximately 50,000 individuals in the US population. To ensure ethical standards, the Research Ethics Review Board at the National Centre for Health Statistics provided approval for the study. The board comprised medical professionals, technicians, and interviewers who conducted surveys, health measurements, and laboratory tests. Prior to their participation, all individuals involved in the study provided informed consent.

**Figure 1:**
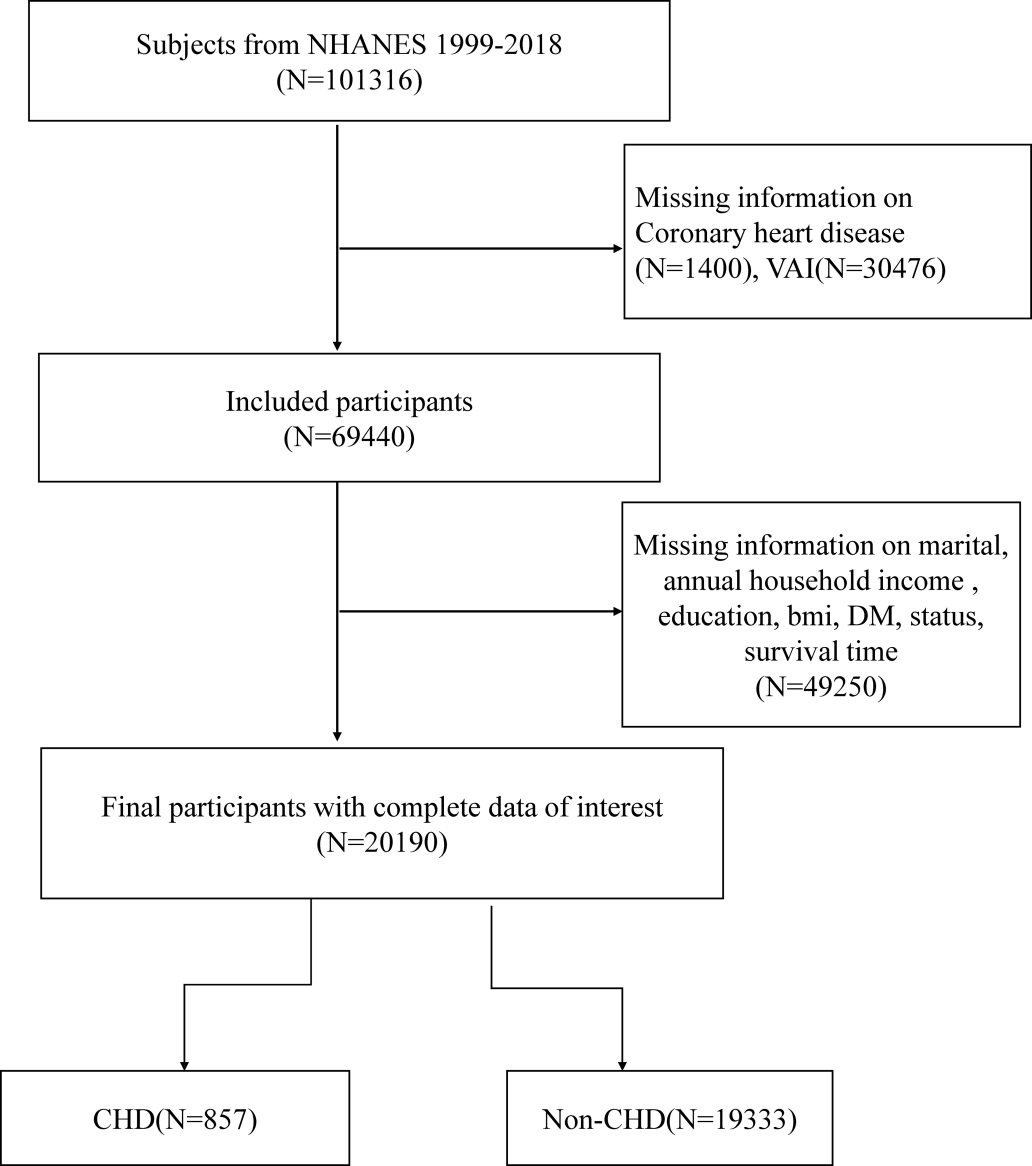
Overview of participants screening. NHANES, National Health and Nutrition Examination Survey; CHD, coronary heart disease; DM, Diabetes mellitus.

NHANES utilized an advanced computer system to collect and process data, enabling researchers to assess disease prevalence and identify risk factors. In this particular study, data from the 1999-2018 NHANES cycle was utilized. The initial cohort comprised 101,316 participants age over 20 years who completed interviews. Exclusions were made for individuals with missing data on coronary heart disease (CHD), VAI, annual household income, education, BMI, diabetes mellitus (DM), status, and survival time (N=81,126). Ultimately, a total of 20,190 participants were included in this cross-sectional analysis. A detailed flow diagram illustrating the participant selection process can be found in the Supporting Information, specifically **Figure 1**.

### VAI score

The VAI score was determined using the equations reported previously (10).

For males, the formula used was:


$$\text{VAI}=\text{WC}(\text{cm})/(39.68+1.88\;\times \text{BMI kg}/{\text{m}}^{2})\text{ ×}(\text{TG}[\text{mmol}/l]/1.03)\text{ ×}{\left(1.31/\text{HDL}-\text{c}[\text{mmol}/l]\right)}^{2}.$$

For females, the formula used was:


$$\text{VAI}=\text{WC}(\text{cm})/(39.58+1.89\text{ ×}\mathrm{BMI kg}/{\text{m}}^{2})\text{ ×}(\text{TG}[\text{mmol}/l]/0.81)\text{ ×}{\left(1.52/\text{HDL}-\text{c}[\text{mmol}/l]\right)}^{2}.$$

To calculate the VAI, NHANES researchers collected anthropometric data including height, weight, calculated BMI, and WC, as well as biochemical data such as glycated hemoglobin, direct HDL-c, and fasting triglycerides (TG). The VAI score provides an estimate of visceral adiposity, with higher scores indicating a greater amount of estimated visceral fat accumulation.

### Ascertainment of outcomes

Participants with CHD were identified based on their positive response to the question, “Has a doctor or other health professional ever told you that you had coronary heart disease?” The participant who reported the problem indicated a medical history of being diagnosed with CHD.

The National Center for Health Statistics gathered mortality data by conducting a probabilistic record match between NHANES participants and the National Death Index (NDI) death certificate data. The NHANES-linked NDI public access data was used until December 31, 2015 to determine both mortality status and the cause of death.

### Covariates

The study considered several factors (covariates) that could potentially impact the relationship between VAI and CHD. These covariates included various demographic characteristics, such as age, sex, ethnicity, marital status, educational level, smoking and alcohol habits, BMI, and annual household income. Moreover, health risk factors like diabetes and hypertension were also taken into consideration. For more detailed categorical data, please refer to **Table 1**.

**Table 1:** Characteristics of the study population.

Variable	Total (N=20190)	Non-CHD (N=19333)	CHD (N=857)	P-value
**Age (Years)**	47.18(0.23)	46.47(0.22)	66.41(0.49)	< 0.0001
**Sex**				< 0.0001
Female	50.68%	51.36%	32.24%	
Male	49.32%	48.64%	67.76%	
**Ethnicity**				< 0.0001
black	10.19%	10.38%	5.00%	
other	19.89%	20.20%	11.54%	
white	69.92%	69.42%	83.46%	
**Marital**				< 0.0001
Married	57.47%	57.07%	68.29%	
SDW	18.04%	17.82%	24.07%	
unmarried	24.49%	25.11%	7.64%	
**BMI (kg/m2)**				< 0.001
<25	31.44%	31.72%	23.72	
25-29.9	33.26%	33.20%	34.89	
≥30	35.30%	35.08%	41.39	
**Annual household income**				0.001
0-19.999$	15.91%	15.75%	20.27%	
20.000-54.999$	36.56%	36.41%	40.60%	
55.000-74.999$	14.09%	14.09%	14.23%	
75.000$	33.44%	33.76%	24.90%	
**Education**				0.05
High school graduate or under	55.48%	55.31%	59.99%	
Some college or above	44.52%	44.69%	40.01%	
**Smoke**				< 0.0001
former	25.49%	24.67%	47.96%	
never	53.02%	53.72%	33.89%	
now	21.49%	21.61%	18.15%	
**Alcohol consumption**				< 0.0001
No	30.35%	29.92%	42.24%	
Yes	69.65%	70.08%	57.76%	
**DM**				< 0.0001
DM	14.40%	13.46%	40.01%	
IFG	9.24%	9.03%	15.04%	
IGT	5.33%	5.30%	6.17%	
no	71.03%	72.22%	38.77%	
**Hypertension**				< 0.0001
no	62.76%	64.16%	24.54%	
yes	37.24%	35.84%	75.46%	
VAI				
Mean (SE)	2.13(0.03)	2.11(0.03)	2.53(0.11)	< 0.001
Q1[0.087-0.903]	25.49%	25.76%	17.99%	< 0.0001
Q2 (0.903-1.494]	25.40%	25.60%	20.06%	
Q3(1.494,2.513]	24.50%	24.41%	26.90%	
Q4(2.513-135.67]	24.61%	24.23%	35.04%	
**Status**				< 0.0001
Assumed alive	89.65%	90.63%	62.99%	
Assumed deceased	10.35%	9.37 %	37.01%	

VAI, Visceral Adiposity Index; CHD, Coronary heart disease; Q, quartile; DM, Diabetes mellitus; IFG, Impaired fasting glucose; IGT, impaired glucose tolerance; SDW, Separated, Divorced, Widowed; BMI, Body Mass Index.

### Statistical analyses

In order to account for the complex sampling design in the study, weighted analysis was conducted in accordance with NHANES recommendations. The normal group and the CHD group were compared in terms of their baseline characteristics. For continuous variables, weighted student t-tests were utilized, while weighted chi-square tests were employed for categorical variables. To examine the correlation between VAI and CHD, various models were used in a multivariable logistic regression analysis. Restricted cubic spline was used to assess linear association between VAI and CHD.

Model 1 assessed the relationship between VAI and CHD without adjusting for any confounding variables. Model 2, on the other hand, controlled for age and sex as potential confounders. Model 3 went a step further by considering additional factors, such as ethnic, marital status, annual household income, education level, smoking and alcohol consumption, BMI, hypertension, and diabetes. Furthermore, subgroup analysis was conducted based on age(<50,≥50 years) to investigate the association between VAI and different subgroups within CHD.

Evaluation of non-linear relationships between VAI and the risk of CHD development and all-cause mortality, as well as CVD risk, was conducted using the restricted cubic spline method with three nodes. Kaplan-Meier method and the log-rank test was used to construct overall survival (OS) curves with different level of VAI.

The “nhanesR” package was utilized for data extraction and analysis. Statistical significance was determined by setting a significance level of P< 0.05 (two-sided), indicating the presence of statistically significant differences.

## Results

### Characteristics of the study population

Among the 20,190 participants, 49.32% were male, while 50.68% were female (**Table 1**). The prevalence of CHD was 3.54%. It was observed that individuals with CHD were significantly older compared to the normal participants. Moreover, the CHD cases had a higher proportion of people with a smoking history and higher BMI, but a lower proportion with a history of alcohol consumption. Additionally, the prevalence of hypertension and diabetes was higher in the CHD group. Furthermore, the CHD group had a higher percentage of cases recorded as assumed deceased.

### Association between VAI and CHD

The results of the univariate logistic regression model indicated that there was a significant association between VAI and the risk of CHD, with each unit increase in VAI corresponding to a higher risk of CHD (OR 1.03 [95% CI 1.01-1.05], P<0.001). This association remained statistically significant even after accounting for various covariates such as age, sex, ethnicity, marital status, annual household income, education, smoking, alcohol consumption, BMI, diabetes, and hypertension, in the multivariate logistic regression models. In model 2, the OR was 1.04 [95% CI 1.02-1.06, P<0.001], and in model 3, the OR was 1.03 [95% CI 1.00-1.04, P=0.03]. Furthermore, when VAI was evaluated as a categorical variable, individuals in the fourth quartile (Q4) had the highest risk of CHD compared to those in the top quartile of VAI (OR 1.50 [95% CI 1.12-2.01, P=0.01]) (**Table 2**). This analysis was adjusted for the same covariates as mentioned before.

**Table 2:** Multivariate logistic regression models of VAI and CHD.

Visceral Adiposity Index	Model 1		Model 2		Model 3	P-value
	OR (95%CI)	P-value	OR (95%CI)	P-value	OR (95%CI)	
Continuous	1.03(1.01,1.05)	<0.001	1.04(1.02,1.06)	<0.0001	1.02(1.00,1.04)	0.03
Q1	Ref.		Ref.	Ref.	Ref.	
Q2	1.12(0.84,1.50)	0.44	0.99(0.73,1.34)	0.94	0.92(0.67,1.25)	0.58
Q3	1.58(1.16,2.14)	0.004	1.35(0.99,1.84)	0.06	1.18(0.85,1.62)	0.32
Q4	2.07(1.56,2.75)	<0.0001	1.82(1.37,2.43)	<0.0001	1.50(1.12,2.01)	0.01
P-trend	<0.0001		<0.0001		<0.001	

OR, odd ratio; CI, Confidence interval; VAI, Visceral Adiposity Index.

Model 1: adjust for non.

Model 2: adjust for age, sex.

Model 3: adjust for age, sex, ethnic, marital status, annual household income, education, smoke, alcohol consumption, BMI, diabetes, hypertension.

**Figure 2:**
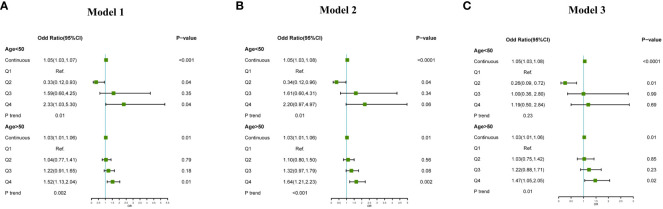
Association between VAI and CHD subgroup analysis by age (<50 years, ≥50 years), **(A)**: adjust for non; **(B)**: adjust for age, sex, ethnic; **(C)**: adjust for age, sex, ethnic, marital status, annual household income, education, smoke, alcohol consumption, BMI, diabetes, hypertension.

In the subgroup analysis based on age, after adjust for confounding factors, a significant positive association was observed between the increase in VAI and the risk of CHD, considering VAI as a continuous variable, for both age groups (<50 years: OR: 1.05, 95%CI:1.03-1.08, P<0.0001;≥50 years: OR: 1.03,95%CI:1.01-1.06,P=0.01). However, when VAI was treated as a categorical variable and adjusting for confounding factors including age, sex, ethnic, marital status, annual household income, education, smoke, alcohol consumption, BMI, diabetes, hypertension, a significant association between high levels of VAI and the increased prevalence of CHD was only observed in the population aged over 50 years (Q4 vs Q1, OR: 1.47,95%CI:1.05-2.05, P=0.02) (**Figure 2**).

**Figure 3:**
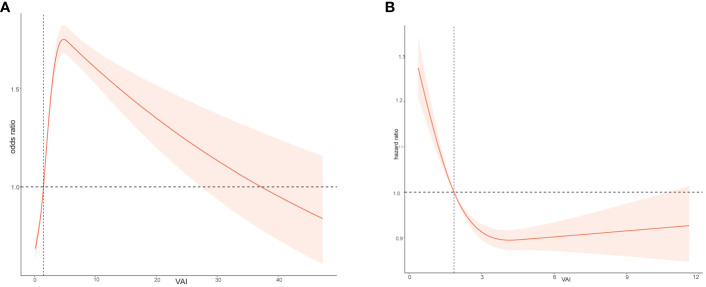
Relation of body Visceral Adiposity Index (VAI) with risk of CHD **(A)** and all-cause death **(B)**.

The relationship between VAI and the OR for CHD was found to be non-linear, as shown using the restricted cubic spline model. The p-value for nonlinearity was less than 0.05, indicating a significant non-linear association between VAI and the risk of CHD (**Figure 3A**). The risk of CHD quickly increases with the rise of VAI and then gradually decreases.

### VAI level and survival outcome

**Figure 4:**
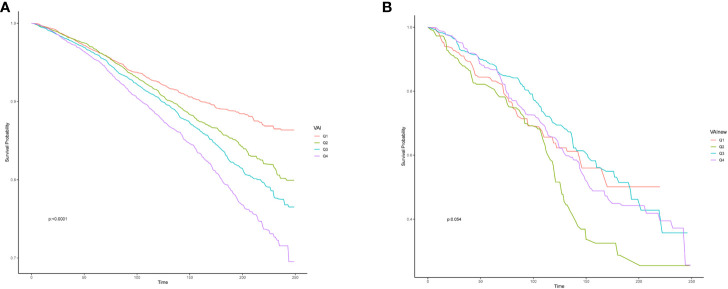
Effect of the Visceral Adiposity Index on overall survival among all population **(A)** and participant with CHD **(B)**, CHD, coronary heart disease.

When investigating the relationship between VAI and overall survival rate in the study population, it was found that there is a non-linear relationship between VAI levels and HR (hazard ratio) (**Figure 3B**). As VAI levels increase, the prognosis for the study population becomes worse. **Figure 4** showed the survival probability of all study populations with different levels of VAI, and it was observed that individuals with higher levels of VAI had significantly poorer prognosis compared to those with lower levels (P<0.001) (**Figure 4A**). There were no statistical differences in survival outcome among population with CHD(*P*=0.054) (**Figure 4B**).

## Discussion

This study examined the association between VAI and CHD using data from a representative national sample. The study demonstrated a non-linear relationship between VAI levels and risk of CHD and overall survival rate. It found that each unit increase in VAI was associated with a higher risk of CHD. This association remained significant even after adjusting for various covariates. Additionally, higher levels of VAI were associated with poorer prognosis and reduced overall survival rate. Age subgroup analysis showed a significant association between high VAI levels and CHD prevalence in individuals aged over 50 years.

Obesity can be categorized into two types: overall obesity and abdominal obesity. However, different obesity phenotypes can result in varying rates of incidence, mortality, and treatment outcomes for coronary heart disease (CHD). While BMI is commonly used as an indicator for overall obesity, it may not be the most suitable measure for individuals with abdominal obesity. This is because there can be cases of “normal weight obesity” within the population, where individuals with a normal weight still carry a higher risk of CVD due to their high WC. Moreover, WC serves as a valuable indicator of abdominal fat and is associated with heart metabolic disorders, cardiovascular diseases, and even mortality. To accurately evaluate abdominal obesity, additional measures such as VAT need to be considered. VAT fat cells exhibit higher metabolic activity and insulin resistance compared to subcutaneous fat cells (SAT). To date, numerous observational studies have explored the association between VAT volume and the risk of coronary artery disease (14–17). Nicklas et al. suggested that VAT served as an independent predictor of myocardial infarction (17). A study conducted by S.J. Kang et al. discovered that the presence of Excess VAT area was linked to the occurrence of coronary stenosis of less than 50% and noncalcified plaques. Importantly, this association was found to be independent of conventional cardiovascular risk factors. The study population consisted of asymptomatic individuals without any prior history of coronary artery disease (18). In addition. Some Mendelian randomization studies had provided further evidence that suggested a causal relationship between VAT and the occurrence of CAD, building upon the foundation of observational research (17, 19). However, it is important to note that a cohort study in older men did not establish a link between VAT and the development of atherosclerotic cardiovascular disease (as defined by CAD death, MI, and fatal or nonfatal stroke) (20). These studies utilized abdominal CT or MRI to measure the VAT. However, these methods are costly, time-consuming, and not commonly employed in clinical settings. An alternative approach involves calculating VAI based on waist circumference, height, weight, as well as TG and HDL-c levels in the bloodstream. This clinical method is straightforward and easily obtainable. Prior studies have indicated that VAI is linked to various health conditions such as diabetes, hyperuricemia, metabolic syndrome, hypertension, atherosclerosis, vascular calcification and heart failure (3, 10, 11, 21). To the best of our knowledge, the current understanding of the relationship between VAI and CHD was still limited. However, our study revealed a significant association between elevated levels of VAI and increased risk of CHD.

As expected, there was a linear relationship between VAI and the OR for CHD. In terms of pathophysiological mechanism, the accumulation of visceral fat in the abdominal cavity increases the load on the heart. Substances secreted by fat cells, such as aldosterone and renin, activate the cardiovascular system, leading to increased blood pressure and blood volume, causing the heart to work harder and increasing coronary blood supply demand (10, 22, 23). Additionally, fat cells secrete inflammatory cytokines such as TNF-α and IL-6, leading to the occurrence of low-grade chronic inflammation (24). These inflammatory cytokines induce endothelial dysfunction and the formation of atherosclerosis, exacerbating the risk of coronary artery narrowing and ultimately leading to coronary heart disease. The presence of visceral fat also disrupts metabolic balance, increasing the risk of metabolic syndrome and diabetes, which further promotes lipid deposition in blood vessels and the development of coronary artery lesions. Moreover, excessive accumulation of fat cells may trigger oxidative stress and endoplasmic reticulum stress, further damaging the heart and promoting the development of coronary artery narrowing.

A recent study conducted by Lee et al. demonstrated that the ratio of visceral-to-subcutaneous fat area (VSR) can serve as a reliable predictor of mortality, irrespective of the total amount of fat present in the body (25). This research highlighted the significance of the distribution of fat deposits rather than the overall fat quantity. Several studies have indicated that as individuals age, the reduction of central fat and fat-free mass may be more influential than BMI in determining the health risks associated with obesity. Additionally, research suggests that the VAI can effectively assess the increased risk of cardiometabolic diseases in patients, particularly elderly women (26). Abdominal obesity, as measured by waist circumference, has also emerged as an independent risk factor for cardiovascular disease, separate from BMI. Advanced imaging techniques have provided valuable insights into body composition, particularly visceral adiposity. These studies emphasize that excess visceral adiposity, including ectopic fat, is indicative of poor cardiovascular outcomes and serves as an important marker for health risks (24). This study supported a strong association between high levels of VAI and poorer survival outcomes. However, no survival differences were observed among individuals with different VAI levels in the population with CHD. A more comprehensive understanding of changes in body composition and fat distribution would help researchers better predict the relationship between obesity, diseases, and mortality rates. The lack of such differences in the population with CHD might be attributed to the relatively small sample size in our study. Future studies might require a larger sample of patients with CHD to validate these findings.

To the best of our knowledge, this is the first large-scale national study in the United States to examine the relationship between VAI and CHD and survival outcomes in a representative adult population. Our research featured a rigorous study design and quality control, a large and representative sample, survival follow-up data, and the advantage of available data on numerous important covariates obtained through integrating NHANES data. Despite these strengths, however, there were still some limitations to this study. Firstly, the NHANES did not collect information on participants regarding echocardiography and coronary angiography, which limited our ability to classify the severity of CHD and conduct sensitivity analyses based on disease classification. Secondly, there was a lack of available data on changes in behaviors such as nutrition, physical activity, sleep, and weakness indicators. Additionally, only all-cause mortality was evaluated. Furthermore, CHD participants were defined as those self-reporting a doctor’s diagnosis of CHD. The same situation occurred for hypertension, diabetes, anemia, liver disease, kidney disease, and history of heart failure. Lastly, this was a cross-sectional study and did not include follow-up data. The changes in VAI and the risk of CHD over time remain unclear. During the study period, our study design does not allow for the determination of a causal relationship between VAI and CHD. More substantial sample sizes or longitudinal studies in the future are needed to further investigate the relationship between VAI, obesity, and CHD.

## Conclusion

This study found a significant non-linear association between VAI and the risk of CHD in a representative sample of the US population. Higher VAI levels were associated with an increased risk of CHD, especially in individuals over 50 years old. Additionally, higher levels of VAI were associated with poorer prognosis and reduced overall survival probability. The findings highlight the importance of monitoring and managing visceral adiposity, as well as implementing preventive interventions to reduce CHD risk. Clinicians should consider age-specific thresholds for VAI and incorporate it into risk assessment and management strategies. These results contribute to a better understanding of the relationship between VAI and CHD and can aid in improving patient outcomes through targeted interventions.

## Availability of data and materials

Publicly available datasets were analyzed in this study. This data can be found here: https://www.cdc.gov/nchs/nhanes.

## Ethics approval and consent to participate

To ensure ethical standards, the Research Ethics Review Board at the National Centre for Health Statistics provided approval for the study. Prior to their participation, all individuals involved in the study provided informed consent.

## Data availability statement

The original contributions presented in the study are included in the article/supplementary material. Further inquiries can be directed to the corresponding authors.

## Author contributions

YaL: Writing – original draft, Writing – review & editing. XZ: Writing – original draft, Writing – review & editing. LC: Conceptualization, Investigation, Writing – original draft. YiL: Writing – original draft, Writing – review & editing. LZ: Supervision, Validation, Writing – review & editing. WC: Supervision, Investigation, Writing – review & editing.

## References

[1] Wu H Chiou J. Potential benefits of probiotics and prebiotics for coronary heart disease and stroke. Nutrients (2021) 13. doi: 10.3390/nu13082878 PMC8401746 34445037

[2] Björkegren JLM Lusis AJ. Atherosclerosis: recent developments. Cell (2022) 185:1630–45. doi: 10.1016/j.cell.2022.04.004 PMC9119695 35504280

[3] Wirtz PH von Känel R. Psychological stress, inflammation, and coronary heart disease. Curr Cardiol Rep (2017) 19:111. doi: 10.1007/s11886-017-0919-x 28932967

[4] Katta N Loethen T Lavie CJ Alpert MA. Obesity and coronary heart disease: epidemiology, pathology, and coronary artery imaging. Curr Problems Cardiol (2021) 46:100655. doi: 10.1016/j.cpcardiol.2020.100655 32843206

[5] NCD Risk Factor Collaboration (NCD-RisC). Worldwide trends in body-mass index, underweight, overweight, and obesity from 1975 to 2016: a pooled analysis of 2416 population-based measurement studies in 128·9 million children, adolescents, and adults. Lancet (2017) 390:2627–42. doi: 10.1016/S0140-6736(17)32129-3 PMC5735219 29029897

[6] Lavie CJ Arena R Alpert MA Milani RV Ventura HO. Management of cardiovascular diseases in patients with obesity. Nat Rev Cardiol (2018) 15:45–56. doi: 10.1038/nrcardio.2017.108 28748957

[7] Lavie CJ De Schutter A Parto P Jahangir E Kokkinos P Ortega FB. Obesity and prevalence of cardiovascular diseases and prognosis-the obesity paradox updated. Prog Cardiovasc Dis (2016) 58:537–47. doi: 10.1016/j.pcad.2016.01.008 26826295

[8] Yusuf S Hawken S Ounpuu S Dans T Avezum A Lanas F. Effect of potentially modifiable risk factors associated with myocardial infarction in 52 countries (the INTERHEART study): case-control study. Lancet (London England) (2004) 364:937–52. doi: 10.1016/S0140-6736(04)17018-9 15364185

[9] Antonio-Villa NE Juárez-Rojas JG Posadas-Sánchez R Reyes-Barrera J Medina-Urrutia A. Visceral adipose tissue is an independent predictor and mediator of the progression of coronary calcification: a prospective sub-analysis of the GEA study. Cardiovasc Diabetology (2023) 22:81. doi: 10.1186/s12933-023-01807-6 PMC10071707 37013573

[10] Bagyura Z Kiss L Lux Á Csobay-Novák C Jermendy ÁL Polgár L. Association between coronary atherosclerosis and visceral adiposity index. Nutrition Metabolism Cardiovasc Dis NMCD (2020) 30:796–803. doi: 10.1016/j.numecd.2020.01.013 32127334

[11] Huang X Jiang X Wang L Chen L Wu Y Gao P. Visceral adipose accumulation increased the risk of hyperuricemia among middle-aged and elderly adults: a population-based study. J Trans Med (2019) 17:341. doi: 10.1186/s12967-019-2074-1 PMC6785935 31601236

[12] Amato MC Giordano C Galia M Criscimanna A Vitabile S Midiri M. Visceral Adiposity Index: a reliable indicator of visceral fat function associated with cardiometabolic risk. Diabetes Care (2010) 33:920–2. doi: 10.2337/dc09-1825 PMC2845052 20067971

[13] Zhang Y He Q Zhang W Xiong Y Shen S Yang J. Non-linear associations between visceral adiposity index and cardiovascular and cerebrovascular diseases: results from the NHANES (1999-2018). Front Cardiovasc Med (2022) 9:908020. doi: 10.3389/fcvm.2022.908020 35811709 PMC9263190

[14] Tanaka T Kishi S Ninomiya K Tomii D Koseki K Sato Y. Impact of abdominal fat distribution, visceral fat, and subcutaneous fat on coronary plaque scores assessed by 320-row computed tomography coronary angiography. Atherosclerosis (2019) 287:155–61. doi: 10.1016/j.atherosclerosis.2019.06.910 31295672

[15] Marques MD Santos RD Parga JR Rocha-Filho JA Quaglia LA Miname MH. Relation between visceral fat and coronary artery disease evaluated by multidetector computed tomography. Atherosclerosis (2010) 209:481–6. doi: 10.1016/j.atherosclerosis.2009.10.023 19922936

[16] Kouli GM Panagiotakos DB Kyrou I Georgousopoulou EN Chrysohoou C Tsigos C. Visceral adiposity index and 10-year cardiovascular disease incidence: The ATTICA study. Nutrition Metabolism Cardiovasc Dis NMCD (2017) 27:881–9. 10.1016/j.numecd.2017.06.015 28851556

[17] Nicklas BJ Penninx BW Cesari M Kritchevsky SB Newman AB Kanaya AM. Association of visceral adipose tissue with incident myocardial infarction in older men and women: the Health, Aging and Body Composition Study. Am J Epidemiol (2004) 160:741–9. doi: 10.1093/aje/kwh281 15466496

[18] Kang SJ Kim D Park HE Choi SH Choi SY Lee W. Visceral adipose tissue area is associated with coronary stenosis and noncalcified plaques. Int J Obes (2005) . 2014 (38):272–8. doi: 10.1038/ijo.2013.105 23748189

[19] Chen Q Wu Y Gao Y Zhang Z Shi T Yan B. Effect of visceral adipose tissue mass on coronary artery disease and heart failure: A Mendelian randomization study. Int J Obes (2005) 2022. (46):2102–6. doi: 10.1038/s41366-022-01216-x 35995978

[20] Schousboe JT Kats AM Langsetmo L Vo TN Taylor BC Schwartz AV. Central obesity and visceral adipose tissue are not associated with incident atherosclerotic cardiovascular disease events in older men. J Am Heart Assoc (2018) 7:e009172. 30369326 10.1161/JAHA.118.009172 PMC6201395

[21] Zhang X Sun Y Li Y Wang C Wang Y Dong M. Association between visceral adiposity index and heart failure: A cross-sectional study. Clin Cardiol (2023) 46:310–9. doi: 10.1002/clc.23976 PMC10018101 36651220

[22] Neeland IJ Poirier P Després JP. Cardiovascular and metabolic heterogeneity of obesity: clinical challenges and implications for management. Circulation (2018) 137:1391–406. doi: 10.1161/CIRCULATIONAHA.117.029617 PMC5875734 29581366

[23] Fox CS Massaro JM Hoffmann U Pou KM Maurovich-Horvat P Liu CY. Abdominal visceral and subcutaneous adipose tissue compartments: association with metabolic risk factors in the Framingham Heart Study. Circulation (2007) 116:39–48. doi: 10.1161/CIRCULATIONAHA.106.675355 17576866

[24] Powell-Wiley TM Poirier P Burke LE Després JP Gordon-Larsen P Lavie CJ. Obesity and cardiovascular disease: A scientific statement from the american heart association. Circulation (2021) 143:e984–e1010. doi: 10.1161/CIR.0000000000000973 33882682 PMC8493650

[25] Lee SW Son JY Kim JM Hwang SS Han JS Heo NJ. Body fat distribution is more predictive of all-cause mortality than overall adiposity. Diabetes Obes Metab (2018) 20:141–7. doi: 10.1111/dom.13050 28671751

[26] Bosello O Vanzo A. Obesity paradox and aging. Eating Weight Disord EWD (2021) 26:27–35. 10.1007/s40519-019-00815-4 31865598

